# Effects of Different Local Moxibustion-Like Stimuli at Zusanli (ST36) and Zhongwan (CV12) on Gastric Motility and Its Underlying Receptor Mechanism

**DOI:** 10.1155/2015/486963

**Published:** 2015-07-12

**Authors:** Yang-Shuai Su, Juan-Juan Xin, Zhao-Kun Yang, Wei He, Hong Shi, Xiao-Yu Wang, Ling Hu, Xiang-Hong Jing, Bing Zhu

**Affiliations:** Institute of Acupuncture and Moxibustion, China Academy of Chinese Medical Sciences, 16 Nanxiaojie, Dongzhimennei, Beijing 100700, China

## Abstract

The aim of this study was to explore the “intensity-response” relationship in local moxibustion-like stimuli- (LMS-) modulated gastric motility and its underlying receptor mechanism. Based on the thermal pain threshold (43°C), 41°C, 43°C, and 45°C LMS were separately applied to ST36 or CV12 for 180 s among ASIC3 knockout (ASIC3−/−) mice, TRPV1 knockout (TRPV1−/−) mice, and their homologous wild-type C57BL/6 mice (*n* = 8 in each group). Gastric motility was continuously measured by an intrapyloric balloon, and the amplitude, integral, and frequency of gastric motility during LMS were compared with those of initial activities. We found that both 43°C and 45°C LMS at ST36 induced significantly facilitated effect of gastric motility (*P* < 0.05), while LMS at CV12 induced inhibited effects (*P* < 0.05). 41°C LMS had no significant impact on gastric motility. Compared with C57BL/6 mice, the facilitatory effect at ST36 and inhibitive effect of LMS at CV12 were decreased significantly in TRPV1−/− mice (*P* < 0.05; *P* < 0.01) but not changed markedly in ASIC3−/− mice (*P* > 0.05). These results suggest that there existed an “intensity-response” relationship between temperature in LMS and its effects on gastric motility. TRPV1 receptor played a crucial role in the LMS-modulated gastric motility.

## 1. Introduction

Acupuncture and moxibustion, also known as “acupmoxa,” were commonly combined to treat a wide range of disorders in oriental countries. The neural mechanism of acupuncture has been widely accepted to activate various afferent fibers, including A*β*-, A*δ*-, and C-fibers, so as to achieve its regulatory functions [1]. The activation of different afferent fibers could produce distinct effects on certain conditions [2–4]. Our previous work showed that modulatory effects of electroacupuncture on gastric motility were closely related to the stimulating intensity, which the A*δ* fiber and TRPV1 receptor were involved in [5].

Moxibustion is a technique that applies heat to acupoints by burning compressed powdered herbal material. During the last decade, an increasing number of clinical and experimental settings on moxibustion have been conducted. The therapeutic effectiveness of moxibustion on various diseases has been observed in humans and animals, including knee osteoarthritis [6], breech presentation [7], stroke rehabilitation [8], and gastrointestinal disorders in terms of improving gastrointestinal motility, protecting gastric mucosa, and relieving visceral hyperalgesia [9–12]. Further studies indicated that temperature-related (local somatothermal stimulation, LSTS) and non-temperature-related factors (smoke, odor, and herbs) are likely to be involved in the mechanism of moxibustion treatment [13]. Moreover, the temperature of thermal stimulation was even emphasized and used as an alternative method of moxibustion in a lot of experimental studies [14, 15].

Although the therapeutic potential of moxibustion to treat gastrointestinal disorders was partially confirmed, the possible “intensity-response” relationship between the temperature of moxibustion and its effects still remains elusive. Which kind of afferent fibers contributes most to the regulatory effect of moxibustion on gastric motility was rarely investigated. Acid sensing ion channel 3 (ASIC3) is a member of the DEG/ENaC family which is known to mediate acid and mechanical responsiveness and located mainly in A*β*-fibers innervating the skin and muscle [16]. Transient receptor potential vanilloid 1 (TRPV1) belongs to TRPV subfamily, which is expressed in sensory A*δ*- and C-fibers. TRPV1 can be activated by capsaicin, noxious heat (>42°C), low PH, and voltage and closely related to noxious physical detection [17]. How a different temperature of moxibustion affects the gastric motility arouses our interest. In the present study, based on the thermal pain threshold (43°C), LMS with different intensities of temperature were introduced to reveal the “intensity-response” manner in LMS-modulated gastric motility. Meanwhile, both ASIC3 gene knockout (ASIC3−/−) mice and TRPV1 gene knockout (TRPV1−/−) mice were employed to establish deletion of A*β*-fiber and A*δ*-/C-fibers models, respectively, in order to explore the distinct roles of A*β*-fiber and A*δ*-/C-fibers and the underlying receptor mechanism during the LMS-modulated gastric motility.

## 2. Materials and Methods

### 2.1. Animal Preparation

Male ASIC3−/− mice (*n* = 8), TRPV1−/− mice (*n* = 8), and C57BL/6 mice (*n* = 8), weighing 20–25 g, were purchased from Jackson Lab (US) and bred at the China Academy of Chinese Medical Science Animal Care Facility. The animals were housed under a natural diurnal cycle at room temperature with free access to food and water. The tail tips of ASIC3−/− and TRPV1−/− mice were cut off after experiment, and genome DNA extracted from mice tails was subjected to polymerase chain reaction (PCR) for further genotype identification to make sure that only homozygous mice were involved. The experiments were conducted in accordance with the Guide for Use and Care of Medical Laboratory Animals from Ministry of Public Health of People's Republic of China.

### 2.2. Gastric Motility Recording

After an overnight fast, the animals were anesthetized with 10% urethane (1.0–1.2 g/kg, via intraperitoneal route). Surgical procedures were carried out as described previously [5]. Briefly, a tracheotomy was performed to maintain an open, low resistance airway. A longitudinal incision was made in the duodenum 0.5 cm distal to the pylorus. A small balloon made of flexible condom rubber was inserted via the incision into the pyloric area and kept in position by tying the connecting catheter to the duodenum wall. And another catheter (inner diameter of 1 mm) was also inserted through the same incision in order to drain digestive juices secreted from stomach. The balloon was filled with 0.05–0.08 mL warm water, to keep the pressure at about 10 cm H_2_O.

Pressure in the balloon was measured by a transducer through a thin polyethylene tube (1.5 mm in outer diameter) and then transmitted into a polygraph amplifier (NeuroLog, NL900D). The signal was captured online and analyzed offline by a data acquisition system (Power-Lab/4s, AD Instruments) and Chart 5.2 software. Demi-fasting gastric contraction was recorded as a control for at least 30 min before LMS. The body temperature of the mice was maintained at about 37.5°C with a feedback-controlled heating blanket. At the end of experiments, the animals were killed with an overdose of urethane.

The first LMS was applied when gastric motility wave maintained stable, usually at 30 minutes after the surgical procedure. The latter stimulus can only be applied when the gastric motility recovered to control state. Responses of gastric motility in each 30 s during LMS were compared with the initial activity in terms of the average amplitude (the average difference between the cyclic maxima and minima in the selected cycles), integral (calculated as the sum of the data points multiplied by the sample interval), and frequency (per minute) of gastric contraction waves. The initial gastric motility and gastric motility during and after LMS were recorded continuously.

### 2.3. Local Moxibustion-Like Stimuli (LMS)

The LMS was performed by application of a heat generator (Physitemp Controller NTE-2A, Physitemp Instruments Inc., USA) with a probe (1 cm in diameter) connected. The stimulation parameters of the instrument were set at 41°C, 43°C, and 45°C, respectively. Hair located around the acupoints was cut off to expose the local skin before LMS application. When the temperature was stable, the LMS would be given by attaching the probe to the skin area (acupoints) for 180 s. The three distinct LMS were separately applied to mice at ST36 or CV12 in an ascending order.

### 2.4. Statistical Analysis

In each 30 s during LMS, changes in the average amplitude, integral, and frequency of gastric motility were calculated by the following formula: (the value during LMS − the value pre-LMS)/the value pre-LMS × 100%. The normalized data before and during LMS in the same group or a different group were compared statistically by a paired *t*-test or unpaired *t*-test. *P* < 0.05 was considered as a statistical significance. All data are expressed as mean ± SE.

## 3. Results

### 3.1. Baseline Recording of Gastric Motility

The gastric motility was detected by intragastric pressure. Regarding gastric motor characteristics, changes in both intragastric pressure and rhythmic contraction were noteworthy. Generally, the intragastric pressure represents the index of gastric tone motility and rhythmic contraction represents gastric peristalsis induced by circular muscle contractions, similar to slow wave of gastric motor activity. The pressure was maintained at about 10 cm H_2_O as baseline by expanding the volume of the balloon with warm water, and rhythmic contractions were recorded at a rate of 4–6/min with 0.5–2.0 cm H_2_O in amplitude.

### 3.2. Facilitatory Effects on Gastric Motility Induced by LMS at ST36 Required TRPV1 Receptor

**Figure 1:**
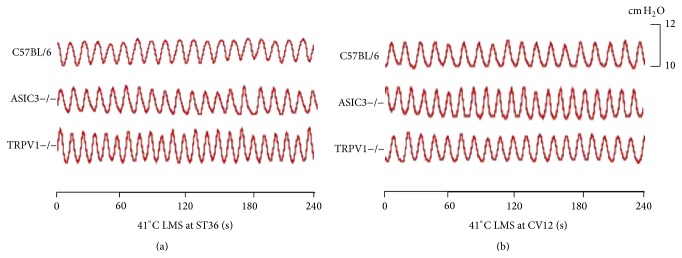
Changes of gastric motility in response to 41°C LMS among three groups of mice. (a) and (b) displayed representative examples of the alterations of gastric contraction wave induced by 41°C LMS at ST36 or CV12, respectively.

**Figure 2:**
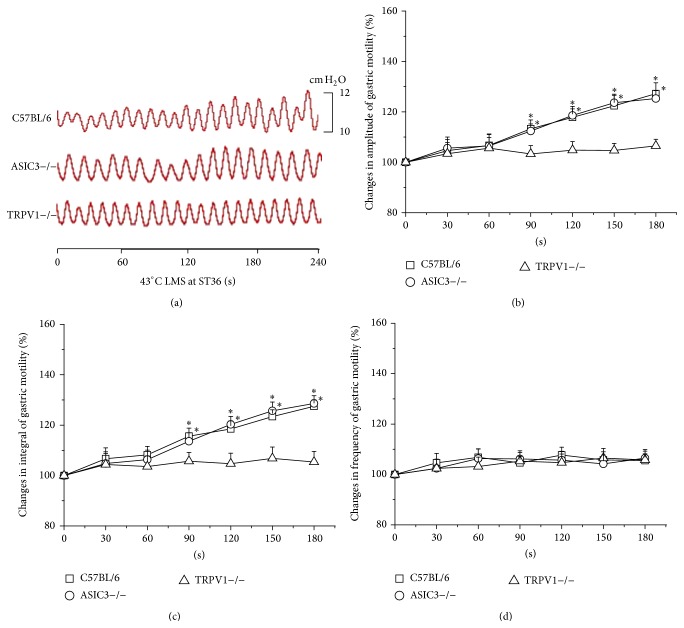
Modulation of gastric motility by 43°C LMS at ST36 among three groups of mice. (a) Representative examples of the alterations of gastric contraction wave induced by 43°C LMS at ST36. (b), (c), and (d) display the pooled data corresponding to the effects of 43°C LMS at ST36 on the amplitude, integral, and frequency of gastric motility in 180 s, respectively. Values before LMS application are presented as 100% and the rates of change are calculated during LMS in each 30 s (^*^ *P* < 0.05, compared with the values before LMS application).

LMS at ST36 produced facilitatory effects on gastric motility which were related to the temperature intensities. Instead of 41°C LMS (Figure 1(a)), 43°C and 45°C LMS at ST36 induced facilitatory effects on gastric motility.  Figure 2(a) showed typical responses of gastric motility due to 43°C LMS at ST36 among C57BL/6, ASIC3−/−, and TRPV1−/− mice. The amplitude and integral of gastric motility were increased markedly from 60 s to 180 s in C57BL/6 mice (amplitude increased by 13.3 ± 3.5% to 27.2 ± 4.3% from 60 s to 180 s, *P* < 0.05, Figure 2(b); integral increased by 15.3 ± 3.2% to 27.5 ± 2.6%, *P* < 0.05, Figure 2(c)). Similarly, ASIC3−/− mice exhibited comparable gastric responses to 43°C LMS (data not shown, *P* < 0.05, Figures 2(b) and 2(c)). However, 43°C LMS at ST36 had no impact on the frequency of gastric motility in both C57BL/6 and ASIC3−/− mice. Intriguingly, 43°C LMS at ST36 failed to produce any significant change in TRPV1−/− mice (Figure 2), suggesting that the responses of gastric motility induced by 43°C LMS at ST36 were mediated by nociceptive primary afferents via TRPV1 receptor.

**Figure 3:**
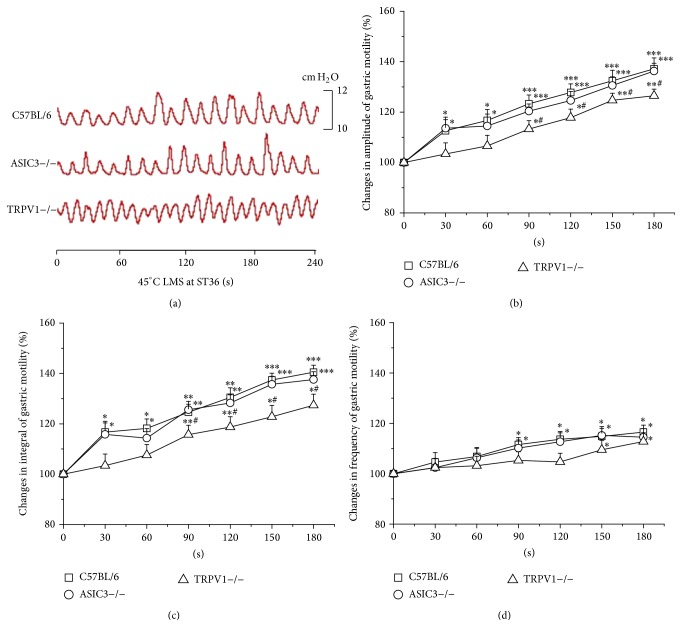
Modulation of gastric motility by 45°C LMS at ST36 among three groups of mice. (a) Representative examples of the alterations of gastric contraction wave induced by 45°C LMS at ST36. (b), (c), and (d) display the pooled data corresponding to the effects of 45°C LMS at ST36 on the amplitude, integral, and frequency of gastric motility in 180 s, respectively (^*^ *P* < 0.05, ^**^ *P* < 0.01, and ^***^ *P* < 0.001, compared with the values before LMS application; ^#^ *P* < 0.05, compared with the facilitatory effect of 45°C LMS at ST36 in C57BL/6 mice).

45°C LMS at ST36 induced more potent modulation on gastric motility (Figure 3). In C57BL/6 and ASIC3−/− mice, 45°C LMS at ST36 produced marked enhancement on the amplitude (increased by 12.6 ± 4.4% to 37.2 ± 4.3% in C57BL/6 mice, *P* < 0.05, *P* < 0.001; 13.6 ± 4.4% to 36.3 ± 3.1% in ASIC3−/− mice, *P* < 0.05, *P* < 0.001, Figure 3(b)) and integral (16.7 ± 4.3% to 40.5 ± 2.8% in C57BL/6 mice, *P* < 0.05, *P* < 0.01, and *P* < 0.001; 15.8 ± 4.6% to 37.6 ± 3.1% in ASIC3−/− mice, *P* < 0.05, *P* < 0.01, and *P* < 0.001, Figure 3(c)) of gastric motility from the first 30 s. However, with a 60 s-latency only moderate increases of the amplitude (*P* < 0.05, *P* < 0.01, Figure 3(b)) and integral (*P* < 0.05, *P* < 0.01, Figure 3(c)) were elicited in TRPV1−/− mice, which were significantly weaker than those in C57BL/6 mice (*P* < 0.05). Besides, latency of the stimulatory effects of 45°C LMS at ST36 on the frequency of gastric motility was shorter in C57BL/6 and ASIC3−/− mice than that of TRPV1−/− mice (Figure 3(d)).

### 3.3. Inhibitory Effects on Gastric Motility Induced by LMS at CV12 Also Required TRPV1 Receptor

**Figure 4:**
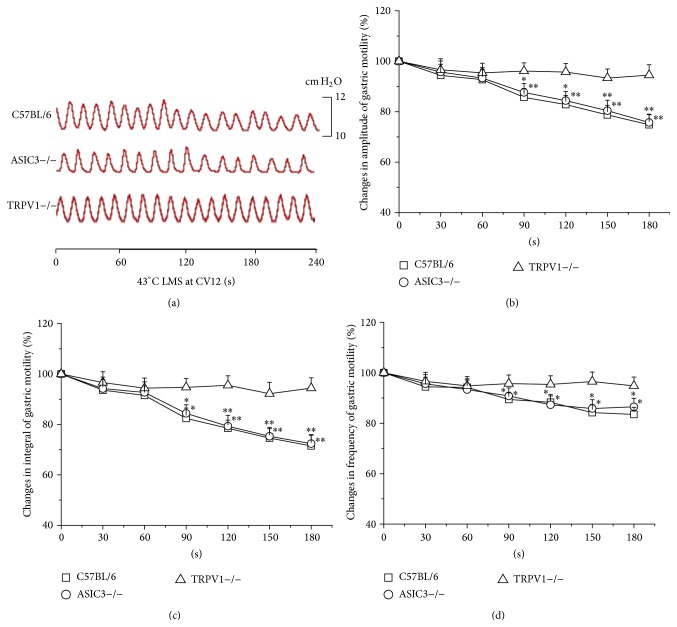
Modulation of gastric motility by 43°C LMS at CV12 among three groups of mice. (a) Representative examples of the alterations of gastric contraction wave induced by 43°C LMS at CV12. (b), (c), and (d) display the pooled data corresponding to the effects of 43°C LMS at CV12 on the amplitude, integral, and frequency of gastric motility in 180 s, respectively (^*^ *P* < 0.05, ^**^ *P* < 0.01, compared with the values before LMS application).

LMS at CV12 elicited inhibitory effects on gastric motility which were dependent on the temperature intensities. Similar to the results of ST36, 41°C LMS at CV12 had no impact on the gastric motility (Figure 1(b)). Nevertheless, as shown in Figure 4, the gastric motility was markedly inhibited in the last 120 s during 43°C LMS at CV12 in C57BL/6 and ASIC3−/− mice (data not shown, Figure 4, *P* < 0.05, *P* < 0.01). Moreover, 43°C LMS at CV12 failed to produce any significant change in TRPV1−/− mice, which reproved the critical role of TRPV1 receptor in 43°C LMS-modulated gastric motility.

**Figure 5:**
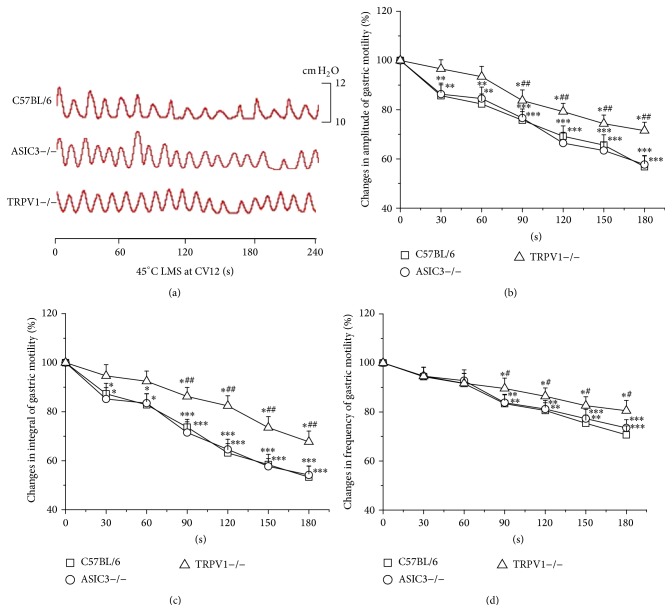
Modulation of gastric motility by 45°C LMS at CV12 among three groups of mice. (a) Representative examples of the alterations of gastric contraction wave induced by 45°C LMS at CV12. (b), (c), and (d) display the pooled data corresponding to the effects of 45°C LMS at CV12 on the amplitude, integral, and frequency of gastric motility in 180 s, respectively (^*^ *P* < 0.05, ^**^ *P* < 0.01, and ^***^ *P* < 0.001, compared with the values before LMS application; ^#^ *P* < 0.05, ^##^ *P* < 0.01, compared with the facilitatory effect of 45°C LMS at CV12 in C57BL/6 mice).

During the application of 45°C LMS at CV12, obvious decreases in the amplitude and integral of gastric motility emerged from the first 30 s of LMS in C57BL/6 and ASIC3−/− mice (*P* < 0.05, *P* < 0.01, and *P* < 0.001) while occurring in the last 120 s in TRPV1−/− mice (*P* < 0.05, Figures 5(b) and 5(c)). In addition, the frequency of gastric motility was markedly decreased by 45°C LMS at CV12 in the last 120 s among the three groups of mice (*P* < 0.05, *P* < 0.01, and *P* < 0.001, Figure 5(d)). Furthermore, the inhibitory effects on gastric motility induced by 45°C LMS at CV12 in TRPV1−/− mice were significantly lower than those in C57BL/6 mice (*P* < 0.05, *P* < 0.01).

## 4. Discussion

In the present study, the “intensity-response” relationship between LMS and its effects on gastric motility was investigated. And the involvement of TRPV1 receptor in the effect of LMS on gastric motility by using of gene knockout mice was firstly observed. Our results revealed the existence of the “acupoint-specific” and “intensity-response” manners in LMS-modulated gastric motility. Both facilitatory effect of ST36 and inhibitive effect of CV12 induced by LMS were dependent on the temperature of LMS by which afferent fibers were activated. Except for 41°C, 43°C and 45°C LMS induced significant modulation on gastric motility in mice. Additionally, in comparison with C57BL/6 mice, the facilitatory effect of LMS at ST36 and inhibitive effect of LMS at CV12 were not changed markedly in ASIC3−/− mice (*P* > 0.05), but these effects decreased significantly in TRPV1−/− mice (*P* < 0.05). Taken together, these results suggested that LMS-modulated gastric motility was mediated by TRPV1 heat nociceptor, which was expressed mainly in A*δ*-/C-fibers and activated by >42°C thermal stimulus, whereas the innoxious warm stimulus could hardly elicit this somatovisceral reflex.

Previous studies showed that local somatothermal stimulation (LSTS) on different acupoints could regulate the function and motility of visceral organs. Chiu and his colleagues found that LSTS at designated acupoints relaxed the sphincter of oddi and the anal sphincter via the neural release of nitric oxide (NO) [18, 19]. In addition, the protective effect of LSTS-induced heat shock protein 70 (HSP70) on relative organs against ischemia-reperfusion (IR) injury was also observed in their studies [20, 21]. Recently, the impact of moxibustion temperature on blood cholesterol level has been investigated. Wang et al. found that 46°C moxibustion at ST36 and CV8 significantly decreased the serum cholesterol level in acute hyperlipidemia C57BL/6 mice, but 38°C moxibustion failed to produce the inhibitory effect. Furthermore, in comparison to C57BL/6 mice, the inhibitory effect of 46°C moxibustion was abolished in TRPV1−/−, which indicated the involvement of TRPV1 receptor in this cholesterol-lowering effect. It should be noted that the temperate-specific manner has been shown in all these studies. Based on the researches above and our present study, the temperature setting for moxibustion effectiveness should be seriously considered in both experimental and clinical studies.

It has been proposed that acupuncture at different parts of the body produces distinct effects through diverse somatoautonomic reflexes; for example, the facilitatory effect of EA at hindlimb on gastric motility was mediated by the parasympathetic excitation, whereas the inhibitory effect of EA on abdomen was induced by the increased sympathetic outflow [22–24]. Consistently, in the present study, site-specific facilitatory or inhibitory effect on gastric motility had also been observed by LMS, which indicated that the neural mechanism involved in these somatovisceral reflexes may be similar, but the definite pathway needed to be explored further.

The transient receptor potential (TRP) family is the most important temperature-activated ion channels [25]. In mammals, thermally sensitive TRPs (mainly including TPRV1, TRPV2, TRPV3, TRPV4, TRPA1, and TRPM8) are each tuned to a different temperature range and most are expressed in cutaneous sensory neurons [26]. TPRV1 receptor highly expressed on nociceptive A*δ*- and C-fibers can be activated by temperature above 42°C, generating an action potential via a robust calcium influx. A previous study showed that TPRV1 knockout weakened heat-activated currents as well as the prolonged latencies of heat-evoked paw and tail withdrawal response in mice [27]. Thus, it is reasonable to speculate that the impulse induced by LMS-activated TRPV1 receptor on A*δ*- and C-fibers will be transmitted to central nerve system to fulfill the regulatory effects of moxibustion. The remaining regulatory effects of 45°C LMS on gastric motility of TRPV1−/− mice observed in the present study might be contributed to the possibility of the unknown ion channels-mediated heat perception and transduction, or mediated by nonneuroregulation. Notably, the LMS had a moderate effect on the amplitude and integral of gastric motility approximately ranging narrowly from 15% to 40%, and its influence on the frequency was even less. These properties indicated that moxibustion was more likely to be a self-limiting regulation of homeostasis of the body, suggesting that the moxibustion treatment is a safe therapy.

## 5. Conclusion

In summary, there existed an “intensity-response” relationship between LMS and its effects on gastric motility. 43°C LMS-activated TRPV1 receptor was essential to the LMS-modulated gastric motility.

## References

[1] Kagitani F., Uchida S., Hotta H. (2010). Afferent nerve fibers and acupuncture. *Autonomic Neuroscience: Basic and Clinical*.

[2] Zhu B., Xu W.-D., Rong P.-J., Ben H., Gao X.-Y. (2004). A C-fiber reflex inhibition induced by electroacupuncture with different intensities applied at homotopic and heterotopic acupoints in rats selectively destructive effects on myelinated and unmyelinated afferent fibers. *Brain Research*.

[3] Noguchi E. (2010). Acupuncture regulates gut motility and secretion via nerve reflexes. *Autonomic Neuroscience: Basic and Clinical*.

[4] Li Y.-Q., Zhu B., Rong P.-J., Ben H., Li Y.-H. (2007). Neural mechanism of acupuncture-modulated gastric motility. *World Journal of Gastroenterology*.

[5] Su Y.-S., He W., Wang C. (2013). ‘Intensity-response’ effects of electroacupuncture on gastric motility and its underlying peripheral neural mechanism. *Evidence-Based Complementary and Alternative Medicine*.

[6] Lee S., Kim K. H., Kim T.-H. (2013). Moxibustion for treating knee osteoarthritis: study protocol of a multicentre randomised controlled trial. *BMC Complementary & Alternative Medicine*.

[7] Vas J., Aranda-Regules J. M., Modesto M. (2013). Using moxibustion in primary healthcare to correct non-vertex presentation: a multicentre randomised controlled trial. *Acupuncture in Medicine*.

[8] Chen R.-X., Lv Z.-M., Chen M.-R., Yi F., An X., Xie D.-Y. (2011). Stroke treatment in rats with tail temperature increase by 40-min moxibustion. *Neuroscience Letters*.

[9] Tabosa A., Yamamura Y., Forno E. R., Mello L. E. A. M. (2004). A comparative study of the effects of electroacupuncture and moxibustion in the gastrointestinal motility of the rat. *Digestive Diseases and Sciences*.

[10] Bao C.-H., Wu L.-Y., Wu H.-G. (2012). Moxibustion inhibits apoptosis and tumor necrosis factor-alpha/tumor necrosis factor receptor 1 in the colonic epithelium of crohn's disease model rats. *Digestive Diseases and Sciences*.

[11] Wang X.-M., Lu Y., Wu L.-Y. (2012). Moxibustion inhibits interleukin-12 and tumor necrosis factor alpha and modulates intestinal flora in rat with ulcerative colitis. *World Journal of Gastroenterology*.

[12] Zhou E.-H., Liu H.-R., Wu H.-G. (2009). Suspended moxibustion relieves chronic visceral hyperalgesia via serotonin pathway in the colon. *Neuroscience Letters*.

[13] Chiu J.-H. (2013). How does moxibustion possibly work?. *Evidence-Based Complementary and Alternative Medicine*.

[14] Jiang J.-K., Chiu J.-H., Lin J.-K. (1999). Local thermal stimulation relaxes hypertonic anal sphincter: evidence of somatoanal reflex. *Diseases of the Colon and Rectum*.

[15] Terajima H., Enders G., Thiaener A. (2000). Impact of hyperthermic preconditioning on postischemic hepatic microcirculatory disturbances in an isolated perfusion model of the rat liver. *Hepatology*.

[16] Li W. G., Xu T. L. (2011). ASIC3 channels in multimodal sensory perception. *ACS Chemical Neuroscience*.

[17] Jara-Oseguera A., Simon S. A., Rosenbaum T. (2008). TRPV1: on the road to pain relief. *Current Molecular Pharmacology*.

[18] Chiu J.-H., Lui W.-Y., Chen Y.-L., Hong C.-Y. (1998). Local somatothermal stimulation inhibits the motility of sphincter of Oddi in cats, rabbits and humans through nitrergic neural release of nitric oxide. *Life Sciences*.

[19] Jiang J.-K., Chiu J.-H., Lin J.-K. (2000). Local somatothermal stimulation inhibits motility of the internal anal sphincter through nitrergic neural release of nitric oxide. *Diseases of the Colon and Rectum*.

[20] Lin Y.-H., Chiu J.-H., Tung H.-H., Tsou M.-T., Lui W.-Y., Wu C.-W. (2001). Preconditioning somatothermal stimulation on right seventh intercostal nerve territory increases hepatic heat shock protein 70 and protects the liver from ischemia—reperfusion injury in rats. *Journal of Surgical Research*.

[21] Chiu J. H., Tsou M. T., Tung H. H. (2003). Preconditioned somatothermal stimulation on median nerve territory increases myocardial heat shock protein 70 and protects rat hearts against ischemia-reperfusion injury. *Journal of Thoracic and Cardiovascular Surgery*.

[22] Tatewaki M., Harris M., Uemura K. (2003). Dual effects of acupuncture on gastric motility in conscious rats. *The American Journal of Physiology—Regulatory Integrative and Comparative Physiology*.

[23] Kametani H., Sato A., Sato Y., Simpson A. (1979). Neural mechanisms of reflex facilitation and inhibition of gastric motility to stimulation of various skin areas in rats. *Journal of Physiology*.

[24] Sato A., Sato Y., Suzuki A., Uchida S. (1993). Neural mechanisms of the reflex inhibition and excitation of gastric motility elicited by acupuncture-like stimulation in anesthetized rats. *Neuroscience Research*.

[25] Ramsey I. S., Delling M., Clapham D. E. (2006). An introduction to TRP channels. *Annual Review of Physiology*.

[26] Dhaka A., Viswanath V., Patapoutian A. (2006). Trp ion channels and temperature sensation. *Annual Review of Neuroscience*.

[27] Caterina M. J., Julius D. (2001). The vanilloid receptor: a molecular gateway to the pain pathway. *Annual Review of Neuroscience*.

